# African-American crack abusers and drug treatment initiation: barriers and effects of a pretreatment intervention

**DOI:** 10.1186/1747-597X-2-10

**Published:** 2007-03-29

**Authors:** Wendee M Wechsberg, William A Zule, Kara S Riehman, Winnie K Luseno, Wendy KK Lam

**Affiliations:** 1 Substance Abuse Treatment Evaluations and Interventions Program, Behavioral Health and Criminal Justice Research Division , RTI International, 3040 Cornwallis Road, Research Triangle Park, NC 27709-2194, USA; 2 Macro International, Inc., 3 Corporate Square NE, Suite 390, Atlanta, GA 30329, USA

## Abstract

**Background:**

Individual and sociocultural factors may pose significant barriers for drug abusers seeking treatment, particularly for African-American crack cocaine abusers. However, there is evidence that pretreatment interventions may reduce treatment initiation barriers. This study examined the effects of a pretreatment intervention designed to enhance treatment motivation, decrease crack use, and prepare crack abusers for treatment entry.

**Methods:**

Using street outreach, 443 African-American crack users were recruited in North Carolina and randomly assigned to either the pretreatment intervention or control group.

**Results:**

At 3-month follow-up, both groups significantly reduced their crack use but the intervention group participants were more likely to have initiated treatment.

**Conclusion:**

The intervention helped motivate change but structural barriers to treatment remained keeping actual admissions low. Policy makers may be interested in these pretreatment sites as an alternative to treatment for short term outcomes.

## Background

Sociocultural factors may pose significant barriers for drug abusers seeking health care or substance abuse treatment. These barriers may be particularly problematic for some African-Americans and other disadvantaged populations. To help reduce the negative behaviors and outcomes associated with substance abuse and dependence, new intervention models need to be developed that specifically address the sociocultural environment of ethnic minorities [1]. Moreover, recent research has recognized the need to enhance understanding of crack cocaine dependence and how crack abusers interact with the substance abuse treatment system [2].

Crack is a cheaper and smokable form of cocaine that became widely available in the mid-1980s, and it continues to be a public health problem in the United States. Crack use is present among all ethnic groups [3], but it is most common among African-Americans residing in low-income inner-city neighborhoods [4-7]. Findings from the 2001 National Household Survey on Drug Abuse (NHSDA) indicated that African-Americans made up 12% of the U.S. population, but they represented 19% of individuals who had used crack in the past year [8]. In addition, crack dependence rates are reported to be higher among African-Americans than among Hispanics or Whites [4]. Furthermore, cocaine-related emergency room episodes and overdose deaths were more common among African-Americans than any other racial/ethnic group [4,9].

The prevalence of crack use among African-Americans only partially explains differential drug use across racial/ethnic groups. Many frequent crack abusers are older, unemployed, without health insurance, susceptible to health risks from their use of cocaine, and more likely to be living in social environments where there is increased risk for crack use [10,11]. Moreover, barriers to treatment among African-Americans who abuse crack have not been well researched, although some findings indicate that African-Americans do not have the resources to enter treatment [12].

Harm-reduction outreach efforts specifically targeting hard-to-reach drug users have been successful in reaching out-of-treatment substance abusers [13]. However, many crack abusers are not accessing drug treatment from specialty programs, such as inpatient or outpatient drug rehabilitation facilities or mental health centers, even when offered free coupons for treatment [14]. Instead, they prefer to seek help from health and social service programs, such as emergency room services or self-help groups [15]. The findings from an earlier North Carolina study targeting out-of-treatment African-American injecting drug users (IDUs) and crack abusers for HIV risk reduction suggest that although IDUs can access methadone treatment, crack abusers have more difficulty accessing treatment services [16].

Individual-level barriers to treatment entry also exist, such as motivation and treatment readiness. Motivation for treatment has been conceptualized in a three-stage model consisting of problem recognition, desire for help, and readiness for treatment [17]. Research has shown that problem recognition is a key step in treatment entry [18,19]. Despite its importance, relatively few studies have examined motivation among out-of-treatment African-American crack abusers and its relationship to treatment initiation. One study examined differences between African-American crack abusers who reported being ready for treatment in the next 30 days and those who were not ready. The findings indicated that treatment readiness was significantly associated with problem recognition [20]. Thus, self-recognition that drug use is a problem may be a critical first step in the treatment initiation process.

Even when minority crack abusers want treatment, various external barriers may prevent them from gaining access. The extent of their drug use, economic liabilities, and the drug-user lifestyle may be determining factors, but additional structural barriers – such as cost, transportation, cultural competency, daycare, gender bias – also keep treatment programs from reaching and retaining these populations in their programs [13,21].

The demands of drug treatment programs are often based on models that lack cultural sensitivity to minorities or women. For example, some people may find aspects of the initial involvement in these programs – such as self-disclosure, trust in virtual strangers, being urged to "surrender" or admit they are "powerless" – to be alien and culturally inappropriate. Therefore, if African-Americans are tentative about seeking treatment, outreach referral and intake need to be conducted in a culturally congruent manner [22].

A number of studies support the idea that pretreatment interventions and strategies may be effective in facilitating treatment entry and increasing treatment retention for substance abusers. One survey of studies that have tested various pretreatment strategies concluded that more research was needed to evaluate which strategies or combinations of strategies are most effective [23].

Previous studies have found that for African-Americans there is a need for greater cultural congruence and appropriate consideration of their readiness for treatment, including whether or not they have the confidence and self-efficacy to enter and benefit from substance abuse treatment. To address the needs of African-American crack abusers, a culturally congruent Pretreatment Intervention to enhance treatment motivation and readiness, decrease crack use, and prepare crack abusers for treatment entry was developed and tested. This study examines the effectiveness (at 3- and 6-month follow-ups) of the Pretreatment Intervention compared with a delayed treatment control condition to reduce drug use and encourage treatment initiation and participation.

## Methods

### Outreach and recruitment

Participants were recruited in Raleigh, North Carolina, by indigenous community outreach workers who were themselves in substance abuse recovery. Individuals were screened and recruited according to a prespecified sampling plan through standardized street-outreach techniques, peer-advocate chain referral, and self-referral procedures that have been used in numerous community studies [14,36]. Outreach workers were trained to approach and screen individuals in inner-city neighborhood segments to ensure that the sample comprised multiple communities and social groups. The total number of out-of-treatment African-American crack abusers recruited through these procedures between August 2000 and August 2002 was 567 individuals. Of these, 443 completed the two intake interviews and were randomly assigned to either the intervention or control group.

### Eligibility

Individuals who met eligibility criteria during the screening process on the street were referred to the field site for final determination of eligibility. Participants who met the preliminary criteria indicated that they had an interest in substance abuse treatment and had the intention to reduce or stop drug use within the next 6 months. Other eligibility criteria included self-identifying as African-American, being at least 18 years of age, having no formal substance abuse treatment within the past 90 days, either having a positive urine test for a cocaine metabolite or self-reporting crack use on at least 13 of the past 90 days, and using crack more frequently than injecting drugs.

### Data collection

All study participants who gave informed consent were assessed by self-report at a two-part intake occurring 2 weeks apart, and at 3- and 6-month follow-ups. Each data collection session took about 90 minutes to complete and was conducted using a computer-assisted personal interview (CAPI) protocol. Informed consent and data collection procedures were approved by RTI International's Office of Research Protection and Ethics.

The assessment questionnaires consisted of items and scales drawn from standardized instruments, including the Global Appraisal of Individual Needs (GAIN) [37] and the Texas Christian University (TCU) Treatment Motivation Scales [38], as well as items developed specifically for this study by the research team. The instruments were pilot tested prior to study implementation.

The domains assessed at intake included the following: sociodemographic characteristics, basic needs, historical and current alcohol and other drug use, history of substance abuse treatment, barriers to treatment, motivation, readiness, sexual behavior, physical and mental health, interpersonal violence, criminality, interpersonal relationships, and social support. Follow-up assessments focused on changes in the above-mentioned domains. Participants received $10 compensation at intake 1, $15 at intake 2, and $25 and $30 for the 3- and 6-month follow-up interviews, respectively. Drug use was verified by urinalysis (using Roche Diagnostic System's OnSite/On Trak™) for alcohol, marijuana, opioids, and cocaine metabolites at intake 1 and at 3- and 6-month follow-ups. After completing intake 1, participants attended an HIV pretest counseling session and were offered HIV antibody testing. HIV posttest counseling was scheduled to coincide with intake 2 approximately two weeks later.

### Intervention

Following the second intake session, participants were randomly assigned to one of two study conditions: the Pretreatment Intervention group or a delayed treatment control group. The Pretreatment Intervention aimed to provide African-American crack abusers with personalized feedback about their drug use and associated problems, information about the treatment process, and skills to enhance individual responsibility to reduce drug use. The intervention also provided assistance in developing appropriate social support systems in an environment in which they feel safe and accepted.

It is likley that African-American crack abusers would have a variety of preexisting conditions that could impact their readiness for treatment and that there would be substantial differences between individuals regarding these conditions. Within this context, the Pretreatment Intervention emphasizes a supportive environment for African-American participants, education in the process of becoming a substance abuse treatment patient, and help in understanding the concepts of recovery. The intervention paradigm supports the conceptual framework with a four-stage approach consisting of (1) the patient's awareness of a problem, (2) the patient's understanding of how to fix the problem, (3) an open attitude to change on the part of the patient, and (4) behavior or action that shows positive movement [16].

This paradigm forms the basis for translating the conceptual framework into a workable protocol to enhance treatment readiness through a variety of well-known intervention strategies, including motivational interviewing [39], role induction [40-42], and social skills building [43,44]. A combination of intervention strategies is likely to be more effective than a single strategy [23].

The intervention consisted of one individual session and two group sessions conducted over a one-month period. Three African-American individuals (one male and two females) who were indigenous to the community and in substance abuse recovery were hired and trained to deliver the intervention. The sessions were designed to increase treatment readiness and motivation, to assist in reducing external barriers to treatment entry, and to increase understanding of the patient's role in treatment induction and the treatment process. The individual session immediately followed intake 2, and the group sessions were scheduled to occur within 2 weeks of the prior session.

During the 90-minute individual session, the interventionist used motivational interviewing techniques to review individual problem areas with the participant, including barriers to treatment that he or she reported during the data collection interviews. This personalized assessment of drug use, sexual risk, and treatment history allowed participants to develop an individualized plan of action to reduce risk behaviors and enter drug treatment based on their unique life situation. Personal plans focused not only on drug treatment and risk behaviors, but also on life issues such as education, employment, housing, and parenting.

The one-hour group sessions used a support-based format to help participants understand how they are affected by the multiple contextual influences in their lives, and to teach portable skills to reduce risk and increase problem-solving skills and awareness of options for treatment entry. Some of the methods used included group role-playing and rehearsal of new skills as a means to experience new social skills and to increase self-efficacy.

Topics discussed during the group sessions included consequences of drug use, warning signs for increased use (e.g., visiting hangouts), developing healthy lifestyle behaviors (e.g., sober friends, clean housing, positive support systems), assertive responses (e.g., learning to respond to triggers by role-playing and rehearsing assertive and proactive responses to anticipated scenarios), dealing with the return of obsessive use (relapse) as an opportunity for learning, and relationship and prevention enhancements. Participants also received information for developing support networks and linkages to social services. Group sessions also sought to help participants clarify what it means to be in treatment, what treatment can offer them, and what they must be prepared to do.

The study's main hypothesis postulated that the Pretreatment participants would be more likely to initiate treatment and enter treatment. The control group received no intervention during the first 6 months of study enrollment. At 6-month follow-up, individuals in the control group were invited to participate in the Pretreatment Intervention: 10% of study participants (n = 22) attended both the individual session and at least one group session.

### Study sample

The experimental sample comprises 443 male and female out-of-treatment crack abusers who completed both Part 1 and Part 2 of the baseline interview, were randomly assigned to the intervention or control group, and had complete data for the key variables of interest. Comparisons were made on demographics and drug use between individuals who completed both Part 1 and Part 2 (the current sample), and the 104 individuals who completed only Part 1 of the baseline. There were no significant differences in gender, age, education, homelessness, daily crack use, or daily alcohol use. Analyses of the intervention effect included individuals who were randomly assigned and completed either the 3-month or the 6-month follow-up. Ninety percent of the sample completed the 3-month follow-up (n = 400) and 89% completed the 6-month follow-up (n = 396). Because of the low attrition rate, baseline statistics are reported for the entire baseline sample. Table 1 presents the background characteristics of the sample.

**Table 1:** Background Characteristics of Study Sample at Baseline

**Characteristic**	**All Participants (N = 443)**
**Sociodemographic**	
% Male	73.1
Mean age (S.D.)	39.9 (7.8)
% Married or living with partner	16.3
% High school graduate	50.1
% Employed full time	32.0
% Currently homeless	38.1
% Have any type of health insurance	21.3
% Any type of criminal justice involvement	15.1
	
**Drug Use, Treatment History**	
Mean number days smoked crack past 30 days (S.D.)	15.3 (10.4)
% Used alcohol daily past 30 days	32.8
Mean number days drank 5+ drinks past 30 days (S.D.)	11.6 (11.5)
% Used crack daily past 30 days	19.4
Mean number years crack use (S.D.)	13.1 (6.8)
% Ever in drug treatment	59.6
Mean number treatment episodes (S.D.)	1.6 (2.5)
	
**Motivation, Readiness to Change**	
Mean Problem Recognition scale score (S.D.)	8.7 (2.8)
Mean Desire for Help scale score (S.D.)	6.2 (1.3)
Mean Treatment Resistance Index scale score (S.D.)	2.1 (1.4)
% in Preparation Stage of Change – alcohol use	71.6
% in Preparation Stage of Change – crack use	84.2
% in Preparation Stage of Change – treatment entry	64.3
	
**Barriers to Treatment**	
% Transportation	69.7
% Childcare	8.6
% Scheduling around work, school, or family responsibilities	42.1
% Paying for treatment	75.1
% Religious, ethnic, or cultural issues	10.4
	
**Psychological Symptoms**	
Mean depression scale score (S.D.)	2.3 (2.0)
Mean anxiety scale score (S.D.)	1.1 (1.2)

### Measures

#### Outcome measures

Four primary outcomes were examined. Treatment entry at the 3-month and 6-month follow-up was assessed by the question, "During the past 90 days, did you enter a drug treatment program?" This was coded as a dichotomous variable (yes-no). Treatment initiation at the 3-month follow-up was assessed from three items that asked respondents if they made an appointment with a drug treatment program, tried to enter a treatment program, or entered a treatment program in the past 90 days. A yes response to any of these items was considered treatment initiation. This was coded as a dichotomous variable (yes-no). Treatment initiation at 6 months was measured using these three items from the 3-month and the 6-month follow-ups. If individuals responded positively to any of these three items at the 3-month or the 6-month follow-up, they were coded as yes for the 6-month follow-up. Crack use at baseline, 3-months, and 6-months was measured as the number of days crack was used in the past 30 days. Three measures of alcohol use were examined. Alcohol frequency was measured as the number of days of alcohol use in the past 30 days. Alcohol quantity was measured as the number of days an individual drank five or more drinks in one day in the past 30 days, and the number of drinks per day in the past 30 days. Table 2 presents the variable definitions and reliability scores for the measures.

**Table 2:** Variable Definitions and Reliability Scores

**Measure**	**Definition**	**Response Category**	**Chronbach alpha**
**Treatment Motivation**			
Problem Recognition	Awareness of drug problems as measured by items adapted from the Problem Recognition scale of the TCU Motivation for Treatment Scales [38]	9 items Yes = 1, No = 0	0.82
Desire for Help	Awareness of intrinsic need for change in drug use and interest in getting help is measured by items adapted from the Desire for Help scale of the TCU Motivation for Treatment Scale [38]	7 items Yes = 1, No = 0	0.72
Treatment Resistance	Perception of difficulties in being in treatment and resisting use [37]	4 items Yes = 1, No = 0	0.47
Lifetime Treatment Episodes	Reported number of times been to treatment in lifetime	1 item Continuous	
Readiness for Treatment	Desire to go to drug treatment, and how soon individual would want to go	2 items *Precontemplation *= Do no want to or want to go 6+ months from now *Contemplation *= Want to go 1–6 months from now *Preparation *= Want to go in next 30 days	
Readiness to Change Alcohol Use	Desire to change alcohol use	2 items (same categories as above)	
Readiness to Change Crack Use	Desire to change crack use	2 items (same categories as above)	
Years of Crack Use	Number of years since first crack use to baseline	1 item Continuous	
**Psychological Functioning**			
Depressive Symptoms	Significant problems with depressive symptoms reported in the past 90 days adapted from the GAIN Depressive Symptoms Scale [37]	5 items Yes = 1, No = 0	0.88
Anxiety	Significant problems with symptoms of anxiety reported in the past 90 days adapted from the GAIN Anxiety Symptom Index [37]	3 items Yes = 1, No = 0	0.77

#### Covariates

Several demographic measures were included as covariates. Age was entered as a continuous variable; gender was included as a dichotomous variable, with male coded as 1 and female coded as 0. Treatment readiness was entered as a categorical variable that was constructed from two other variables: (1) Do you want to go to treatment? (yes-no); and (2) How soon do you want to go to treatment? (within next 30 days, next 1–6 months, not within the next 6 months).

### Analyses

Analyses of changes in crack and alcohol use compared the within-group differences in the means of the intervention and control group between baseline and 3-month follow-up, and baseline and 6-month follow-up. Statistical significance of changes in crack and alcohol use was assessed using paired t-tests.

The effect of the intervention assignment on treatment initiation at 3 and 6 months was estimated and tested using multiple logistic regression, with age, gender, and treatment readiness at baseline entered as covariates.

To assess comparability, the intervention and control groups were compared on baseline demographic variables and other potentially important variables. There were several significant group differences. The intervention group reported significantly more alcoholic drinks per day in the previous 30 days compared with the control group, (mean = 9.3, S.D = 11.1 versus mean = 6.7, S.D = 7.3, p = 0.009; t = -2.6, df = 439), scored lower on the treatment resistance index (mean = 2.0, S.D. = 1.3 versus. mean = 2.3, S.D. = 1.5, p = 0.036, t = -0.28, df = 440,), and scored lower on drug use problem recognition, (mean = 8.4, S.D. = 2.9 versus mean = 9.0, S.D. = 2.6, p = 0.032, t = -2.1, df = 440). However, these variables were tested in the multivariate models and did not account for group differences in outcomes.

## Results

Table 3 presents the baseline, 3-month follow-up, and 6-month follow-up data for each of the primary study outcomes.

**Table 3:** Group Means at Baseline, and 3-Month and 6-Month Follow-ups

		**3-Month Follow-up**		**6-Month Follow-up**	
**Variable**	**Baseline**	I (N = 198) C (N = 200)		I (N = 196) C (N = 198)	
**% Entered Treatment Past 90 Days**^a^					
Intervention		7.6		10.0	
Control		5.5		8.7	
					
**% Made appt./tried/entered Treatment**^a^					
Intervention		20.2*		24.2	
Control		12.5		18.3	
					
	Mean (S.D.)	Mean (S.D.)	DF	Mean (S.D.)	DF
					
**# Days Crack Use**^a,b^					
Intervention	15.1 (10.4)	8.5 (9.4)***	196	6.6 (8.9)***	193
Control	15.2 (10.5)	8.1 (9.3)***	199	6.3 (8.5)***	197
					
**# Days Alcohol Use**^a,b^					
Intervention	17.3 (11.9)	12.8 (11.1)***	196	11.5 (10.6)***	195
Control	17.2 (11.8)	11.9 (10.7)***	197	12.0 (10.5)***	197
					
**# Days Drank 5+ Drinks**^c,d^					
Intervention	10.3 (11.5)	6.0 (9.0)***	197	5.7 (9.1)***	195
Control	10.1 (11.7)	6.3 (9.5)***	197	5.9 (8.4)***	197
					
**# Drinks/Day**^c,d^					
Intervention	9.3 (11.4)	4.6 (7.6)***	196	5.2 (9.4)***	194
Control	6.7 (7.4)	4.3 (4.1)***	196	4.6 (4.8)**	196

*p < .05, **p < .01, ***p < .001

^a^Comparisons are between groups

^b^P-values are calculated using Pearson chi-square test

^c^Comparisons are from baseline to 3-month follow-up and from baseline to 6-month follow-up

^d^P-values are calculated using paired t-tests'.

DF = Degrees of freedom

### Crack and alcohol use

Both groups reported very substantial decreases in the mean number of days of crack use between baseline and 3- and 6-month follow-ups – from about 15 days to about 8 days of use in the previous 30 days. There were no statistically significant differences in frequency of crack use between the intervention and control group at any point. Similarly, both groups reported significant decreases in number of days of alcohol use, number of days drank five or more drinks, and number of drinks per day. There were no significant differences between groups on any of these measures.

### Treatment initiation

Very few participants entered a treatment program at either the 3- or 6-month follow-up (Table 3). Only 7.6% of the intervention group and 5.5% of the control group reported entering treatment at the 3-month follow-up. Similarly low numbers are seen for the 6-month follow-up. These group differences were not significant at either time point. However, a significantly greater proportion of participants from the intervention group (20.2%) compared with the control group (12.5%) engaged in treatment initiation, which included calling a program, making an appointment, or entering treatment.

Table 4 presents the results from the logistic regression predicting the odds of treatment initiation at the 3- and 6-month follow-ups. Controlling for readiness for treatment, homelessness and number of lifetime treatment episodes, individuals in the intervention group had significantly higher odds of treatment initiation compared with the control group at the 3-month follow-up (OR = 1.89, Wald chi-square = 6.01, df = 1, p = 0.014). In addition, individuals in higher stages of change, who were homeless at baseline, and who had experienced more lifetime treatment episodes had higher odds of initiating treatment at the 3-month follow-up. At the 6-month follow-up, however, intervention assignment was not significantly associated with treatment initiation.

**Table 4:** Logistic Regression Models for Treatment Initiation at 3-Month and 6-Month Follow-ups

	**3 Month Follow-up OR**	**95% CI**	**6 Month Follow-up OR**	**95% CI**
Group Assignment				
Control (ref)	1.00		1.00	
Intervention	1.89*	(1.04–2.34)	1.48	(0.90–2.42)
Ready to Change – Treatment	2.14**	(1.06–3.73)	1.66**	(1.15–2.39)
Homeless	1.84*	(1.04–3.24)	2.04**	(1.24–3.33)
Lifetime Treatment Episodes	1.16**	(1.04–1.31)	1.14*	(1.03–1.26)

*p < .05, **p < .01 (p-values are based on the Wald chi-square test with 1 degree of freedom; constant is included in the model)

## Discussion

The low rates of treatment entry at 3-month follow-up (7.5% and 5.5% in the intervention and control groups, respectively) and at 6-month follow-up (10.0% and 8.7% in the intervention and control groups, respectively) are disappointing, but they are not surprising. When extrapolated to a one-year period, they are quite similar and possibly higher than the rates of treatment entry in a prospective observational study of crack abusers in which about 13% per year entered treatment [24].

Some factors that may help explain the low rates of treatment entry in the present study include (1) 68% of the sample was unemployed, (2) 79% was uninsured, (3) 75% indicated that they would need help paying for treatment, and (4) 70% reported that they would need help with transportation. Unfortunately, free or subsidized treatment was not readily available during the study period. For most of the period, the primary program to which participants were referred charged an intake fee of $75, and the location of the treatment program was inconvenient for many participants.

Given these circumstances, intervention effects on treatment initiation were examined. Initation was defined as making an appointment, attempting to enter treatment or entering treatment. The finding at 3-month follow-up that participants in the intervention group were significantly more likely to initiate treatment than participants in the control group (20% vs. 12%) suggests that the intervention was moderately successful in increasing motivation for treatment. The relatively large difference between participants in the intervention group who initiated treatment (20%) and those who actually entered treatment (7.5%) suggests a need for structural changes that reduce treatment program barriers. In particular, changes may be needed to address barriers related to the financial cost, which was reported by 75% of participants, transportation reported by 68%, child care reported by 10%, and scheduling difficulties by 42%.

This interpretation is consistent with findings from previous studies that have shown that coupons and other techniques for removing financial barriers to treatment are effective in increasing treatment entry [13,25-27]. Nonetheless, the potential impact of readily available free treatment on treatment entry in the 80% of participants in the intervention that did not initiate treatment is difficult to predict. Self-efficacy theory suggests that more people would attempt to enter treatment if they thought that the attempt would be successful [28]. However, other factors, such as possible perceptions regarding the limited effectiveness of current treatments for crack abusers, may reduce the impact of increased access to treatment.

As with many other intervention studies with out-of-treatment drug users, participants in both groups reported significant decreases in drug and alcohol use and risk behaviors between baseline and follow-up interviews, but differences between groups were not significant [25,29-31]. Moreover, studies reporting negative findings may substantially underestimate the number of studies with negative findings because of publication bias [32-34]. In the studies by Simpson et al. [30] and Stephens et al. [31], for example, the specific causal mechanisms effecting change have proven particularly difficult to disentangle. The substantial reductions in drug use and HIV risk that have been reported by participants in these studies, regardless of intervention condition, raise the possibility that the choice to participate may represent a decision to begin changing behavior.

The present study, in which significant, but similar, decreases in alcohol and other drug use were reported by participants in the control group and the intervention group is no exception. Possible explanations for these findings include the warm atmosphere at the field site, interactions with recovering staff, and the effects of the interview itself. In addition, the intervention effects of interacting with outreach workers outside of the site, which have been summarized previously, may have had an important impact on behavior [35]. From anecdotal data collected at these sites, participants felt that they were part of the study, and the questions that were asked made them begin to want to make positive changes. Despite the fact that data collection and intervention tasks were performed by different staff and interviewers were trained in techniques for reducing socially desirable responses, social desirability cannot be totally ruled out.

As with almost all studies of out-of-treatment drug users, this study suffers from several potential limitations. Changes in drug use and treatment initiation are based on self-reports. Although interviews were conducted by interviewers experienced in working with this population and trained in techniques for minimizing socially desirable responses, some responses may be inaccurate because of faulty memory or intentional misreporting. In addition, although a targeted sampling approach was used to increase generalizability, it is not possible to determine the representativeness of the sample; so caution should be used in generalizing these findings to other groups of crack abusers. Fifty-six participants reported a history of injection drug use, but only reported injecting in the past 30 days. Consequently, caution should used in generalizing these findings to IDUs that use crack. In addition, although there were over 200 participants in the intervention and control groups, given the small percentage of participants entering treatment the study may have had insufficient statistical power to detect small or medium effect sizes.

## Conclusion

The extremely high follow-up rates – about 95% of participants in each of the intervention and control groups completed at least one follow-up and 84% completed both follow-ups – increase confidence in the finding that involvement in the study was directly associated with significant decreases in crack use. Additionally, qualitative interviews with a small number of participants (n = 18) as well as anecdotal reports from field staff strongly suggest that simply coming to the study's field study office was important for participants in the control group. Qualitative data also suggest that many participants in the intervention group felt that the intervention was "treatment" – or at least a satisfactory substitution for it – and that most participants felt they could not commit to treatment given the individual and structural treatment barriers they faced. Nonetheless, both groups reported significant reductions in crack use. However the goal to access treatment for the experimental group was mixed. Although there were significant findings in initiation, access was still problematic due to structural barriers from the treatment programs even for those attempting to enter.

Because crack abuse continues in many poor communities, outreach and pretreatment sites may offer policy makers options for initiating treatment and reducing drug use. Determining the costs of such a site and identifying the essential elements of a brief pretreatment intervention, similar to the drop-in centers of years past, may be an important next step to understanding the feasibility of this strategy to reduce barriers to treatment. It is unclear whether site, staff, intervention, or even instrumentation components have an effect. Positive changes occur, but it remains difficult to disentangle which components are the most critical ones.

## Competing interests

The author(s) declare that they have no competing interests.

## Authors' contributions

WW was the Principal Investigator and conceptualized the study, supervised the field and research activities and all staff, and finalized the manuscript. WZ was the Coinvestigator and organized the data cleanup, constructed data sets, and conducted data analysis and writing. KR conducted data analysis, interpretation, and writing. WL conducted the literature review and background writing. WKKL participated in background writing, editing, and analysis.
